# Vaccination against Cancer or Infectious Agents during Checkpoint Inhibitor Therapy

**DOI:** 10.3390/vaccines9121396

**Published:** 2021-11-25

**Authors:** Tahseen H. Nasti, Christiane S. Eberhardt

**Affiliations:** 1 Emory Vaccine Center, Department of Immunology and Microbiology, Emory University, Atlanta, GA 30322, USA; Christiane.Eberhardt@unige.ch; 2 Center for Vaccinology, University Hospitals Geneva, 1211 Geneva, Switzerland; 3 Center for Vaccinology, Department of Pathology and Immunology, Faculty of Medicine, University of Geneva, 1211 Geneva, Switzerland; 4 Center for Vaccinology, Department of General Pediatrics, Faculty of Medicine, University of Geneva, 1211 Geneva, Switzerland

**Keywords:** cancer vaccines, COVID vaccines, preventive vaccines, therapeutic vaccines, influenza

## Abstract

The use of immune checkpoint inhibitors (ICI) has substantially increased the overall survival of cancer patients and has revolutionized the therapeutic situation in oncology. However, not all patients and cancer types respond to ICI, or become resistant over time. Combining ICIs with therapeutic cancer vaccines is a promising option as vaccination may help to overcome resistance to immunotherapies while immunotherapies may increase immune responses to the particular cancer vaccine by reinvigorating exhausted T cells. Thus, it would be possible to reprogram a response with appropriate vaccines, using a particular cancer antigen and a corresponding ICI. Target populations include currently untreatable cancer patients or those who receive treatment regimens with high risk of serious side effects. In addition, with the increased use of ICI in clinical practice, questions arise regarding safety and efficacy of administration of conventional vaccines, such as influenza or COVID-19 vaccines, during active ICI treatment. This review discusses the main principles of prophylactic and therapeutic cancer vaccines, the potential impact on combining therapeutic cancer vaccines with ICI, and briefly summarizes the current knowledge of safety and effectiveness of influenza and COVID-19 vaccines in ICI-treated patients.

## 1. Introduction

Traditional vaccines against various infectious agents, such as tetanus, measles, and influenza are administered preventively, that is, before exposure or disease. These vaccines consist mostly of immunogenic surface antigens or of inactivated or attenuated organisms, and they elicit mostly humoral immune responses, which confer protection in case of exposure. In contrast, for cancer vaccines the major component of the immune system that required activation is the tumor-specific T cell compartment. Cancer vaccine-induced T cells can be either de novo or an amplification of pre-existing tumor-specific T cells with a broader repertoire that can strengthen pre-existing tumor immunity. In addition, dendritic cells and NK cells can complement the T cell-mediated anti-tumor immune response. For example, activating dendritic cells (DCs) with TLR-9 agonists, enhances CD8 T cell mediated anti-tumor immunity [1]. Although B cells in tumor immunity have not been widely studied, there is mounting evidence that they play a role beyond neutralizing cancer-causing viruses [2].

In the context of tumor diseases, this review summarizes preventive and therapeutic cancer vaccines and their limitations, elucidates the role of combining immunotherapy with cancer vaccines to potentially increase their efficacy, and overviews the state of knowledge regarding risks and benefits in administrating vaccines against infectious agents to patients under ICI treatment. 

## 2. Preventive Cancer Vaccines

Cancer vaccination strategies can be categorized into preventive and therapeutic approaches. The preventive strategy aims to train the immune system to attack cancer-causing viruses upon infection [3]. This has been shown to be effective in preventing some types of solid tumors, such as cervical, oral, anal or vaginal cancers caused by human papilloma virus (HPV) [4,5,6,7,8] or liver cancer following chronic hepatitis B virus (HBV) infection [9].

Tumor cells can express some viral antigens after infection and these vaccine-induced antiviral immune responses might be able to recognize tumor cells [10]. However, the majority of solid tumors are not caused by viral infections but result from genetic, environmental or still unknown and unpredictable factors. Examples of risk factors are smoking, whereby K-ras mutations lead to lung cancer, familial atypical multiple mole–melanoma (FAMMM) syndrome with driver mutations such as N ras or B raf and the subsequent development of skin cancer [11]. The design and use of preventive vaccines in the context of these non-virally induced cancers is challenging and several elements need to be taken into account. First, viral structures are very different from human structures and are easily identified by the immune system as a foreign target, while tumor cells closely resemble the normal healthy cells from which they originate. Hence, chosen antigens for cancer vaccines need to be sufficiently distinct to avoid any cross-reactivity with healthy tissue and thus prevent autoimmune complications. Second, every individual is unique at a molecular level and even tumor cells of similar cancer entities will have distinct cell phenotypes in different individuals, with their own unique tumor surface antigens. Last, it is impossible to predict driver mutations or types of cancer that a currently healthy individual will—or will not—develop. As a result, there are, as of yet, no approved preventive cancer vaccines that can combat non-virally induced cancers, and this review will focus on the challenges of therapeutic cancer vaccines.

## 3. Therapeutic Cancer Vaccines

The strategy of therapeutic cancer vaccines is to administer them once cancer has been detected and to induce immune responses that are directed against tumor cells. Therapeutic cancer vaccines target two major types of antigens, namely, tumor-specific antigens (TSA) and tumor-associated antigens (TAA) (reviewed in [12]). TSA are antigens found only on cancer cells but not on healthy cells, such as neoantigens, that arise mostly from oncogenic driver mutations or from viral antigens expressed after incorporation of the viral genome, e.g., K-ras mutations, p53 mutations, HPV E6/E7 [13]. Neoantigens can result from genetic alterations, leading to several different types of mutations, such as single-nucleotide variants (SNVs), nucleotide insertions or deletions and frame-shift mutations, which lead to the expression of altered proteins that are not present in healthy tissues and unique to malignant cells [12]. TAA on the other hand are present in specific subsets of healthy cells but have higher levels of expression levels in tumor cells. The melanoma-antigen (MAGE) protein family, for example, includes a tyrosinase expressed at high levels in melanoma cells, however, are found at lower levels in testis and melanocytes. However, the latter are immune privileged sites, and the immune system of healthy individuals is thus naïve to MAGE protein. Upon overexpression by melanoma cells, MAGE proteins are recognized by the immune system as foreign, which results in immune activation.

Therapeutic cancer vaccines are often based on cellular, viral, or molecular (peptide, DNA, or RNA) components that are mainly neoantigens [14,15] and vaccines can be autologous or allogeneic, depending on the feasibility, efficiency and the platform used [15,16]. Using TAA as vaccine antigens is a challenge due to their presence on healthy cells, which results in immunological tolerance and a lack of specificity against cancer cells. Autologous cancer vaccines are derived from the patient’s cells—either tumor cells or cells of the innate or adaptive immune system. One example is the dendritic cell vaccine, whereby autologous DCs from the patients’ blood are purified and loaded with neo-antigens or transfected with genes encoding the antigen of interest. The aim of autologous vaccines is to treat active cancer if present, and to maintain remission and avoid relapse after surgery, radiation, or chemotherapy. Several Phase 2 and Phase 3 trials of such cancer cell vaccines are currently ongoing or have been completed, though none has been as effective as traditional vaccines [17]. The major limitations of autologous vaccines include the high production costs and the lengthy standardization of therapy, which is different for each patient. In contrast, allogenic cancer vaccines use non-self cancer cells, and the same vaccine can be administered to different individuals. They are easy to generate and hence, are cost effective [18]. However, one of the biggest disadvantages of allogenic vaccines is that one epitope may be important and mutated in some patients but may not work or be less immunogenic in many others. Numerous allogenic vaccines have been developed but none of them have demonstrated promising result so far.

The majority of clinical trials use cancer vaccines with either autologous or allogeneic antigens (Table 1) in the form of peptides or proteins with incorporated mutations that have been acquired during cell transformation into cancer cells or that have been predicted [19]. These proteins and peptides can be delivered either alone or with the addition of an immune-stimulating adjuvant. Similar to most of the other therapeutic cancer vaccines, these protein or peptide cancer vaccines are in development and are being tested in pre-clinical or clinical trials for safety, immunogenicity and efficacy (reviewed in [20]).

## 4. Challenges of Therapeutic Cancer Vaccines

Unlike preventive vaccine strategies against viral diseases or even some of the cancer types discussed above, therapeutic vaccines face additional challenges. The major problem is presented by the immunosuppressive microenvironment created by the tumor, using various strategies such as low immunogenicity, high tumor burden (reviewed in [40]), secretion of immunosuppressive cytokines, low accessibility of CD8 T cells to the tumor (reviewed in [41]), the presence of regulatory T cells [42], accumulation of myeloid-derived suppressor cells (MDSCs) [43], tumor-associated macrophages (TAMs) [44], low MHC class I expression on cancer cells and T cell exhaustion (reviewed in [45]. Down-regulation of MHC class I molecules on tumor cells, a common mechanism used by these cells to evade the immune system, results in low antigen presentation, and reduced T cell recognition. Highly proliferative tumors outpace the tumor-killing capacities of immune cells. Secretion of immunosuppressive cytokines such as IL-10 and TGF-β by tumor cells and regulatory T cells result in the suppression of CD8 T cell-mediated tumor killing, due to the suppression of IFN-γ from T cells. Furthermore, tumor cells secrete chemokines that result in abnormal vascular systems that either express low levels of ligands that help homing of CD8 T cells, or express ligands for homing receptors that are not expressed by CD8 T cells. In addition, secretion of VEGF by tumor cells promotes angiogenesis, which generates abnormal vasculature, poorly supporting CD8 T-cell infiltration. Furthermore, the enhanced expression of PD-L1 or other inhibitory ligands such as CTLA-4 on cells within the tumor often restrains the effector functions and the activation of T cells [46]. Even if the host body outside the tumor induces a robust immune response to the vaccine by inducing neoantigen-specific T cells, the expression of high levels of checkpoint molecules has been associated with an impaired anti-tumor response. Thus, the inability of the tumor-specific effector immune cells to reach or function inside the tumor microenvironment makes the vaccine ineffective. Furthermore, MDSCs are known to be partially responsible for resistance to therapeutic vaccination despite their important vaccine-mediated activation of T cells in mice and humans [47]. TAMs, with a predominant M2 phenotype [44] and cancer-associated fibroblasts [48], suppress the activation, proliferation and effector function of tumor-specific T cells through the expression of cytokines, chemokines and inhibitory receptors [49,50,51,52]. All these cells significantly affect the efficacy of anti-tumor T cell immunity induced by therapeutic vaccinations. Cancer-associated fibroblasts exert pro-tumor effects by rearranging the extracellular matrix, inhibiting the proliferation and migration of DCs and vaccine-induced T cells, and selectively allowing the infiltration of MDSCs. Therefore, it is crucial to develop combination therapies that improve vaccine efficacy within the tumor microenvironment. One approach that has been demonstrated to be beneficial is the combination of vaccines with immunotherapy.

## 5. Immune Checkpoint Blockade and Combination with Therapeutic Cancer Vaccines

Immune checkpoint inhibitors (ICIs) have shown tremendous promise in treating a variety of cancers, precipitating a new era in cancer immunotherapy. Checkpoint inhibitors increase endogenous anti-tumor activity by blocking components of the immune system, including cytotoxic T lymphocyte-associated antigen 4 (CTLA-4), programmed cell death protein-1 (PD-1), or ligand 1 (PD-L1). These ICIs have revolutionized treatments in more immunogenic cancers such as melanoma, lung cancer, bladder cancer and Hodgkin’s lymphoma [53]. However, despite the clinically promising treatment, the use of mono-therapies is limited. Many patients do not respond to these therapies, or they become resistant over time and require different lines of treatment (reviewed in [54,55]). Furthermore, the results for poorly immunogenic cancers such pancreatic and prostate cancers were discouraging. As described above, limitations of therapeutic cancer vaccines are partially due to tumor immunological phenomena such as T cell exhaustion and immunosuppressive TME with a low proliferation of effector CD8 T cell populations. Thus, combining vaccines and ICI can overcome many of these issues and complement each other for improved clinical outcomes.

### Potential Mechanisms to Increase Cancer Vaccine Efficacy

For example, non-immunogenic tumors such as prostatic and pancreatic cancers are ‘cold tumors’ with limited immune recognition, partially due to a low tumor mutational burden, and they are non-responsive to ICIs [56,57,58,59]. Therapeutic cancer vaccines can not only generate T cells in the periphery, but can also transport these activated tumor-specific T cells into the tumor tissue, thus leading to increased T cell infiltration [60,61]. This can induce tumor cell death with the release of new antigens called “determinant spread’, ‘antigen cascade’ or ‘epitope spreading’ [62] and increase the efficacy of ICI treatment through improved tumor immunogenicity.

As discussed above, one of the important defense mechanisms of progressive tumors, used to counter assaults by the host immune system, is the expression of ligands that bind to immune checkpoint molecules on effector T cells. This leads to diminished cytotoxic killing activity against tumor cells and a decreased secretion of in anti-tumor cytokines such as IFN-γ and TNF-α. This phenomenon, termed “T cell exhaustion”, is a hallmark of tumor resistance to therapeutic vaccine intervention. ICIs reinvigorate exhausted T cells and are used in the treatment of various types of cancers [63]. Since not all patients respond to ICIs, approaches that support a combination of ICIs and cancer therapeutic vaccinations are explored.

ICI can improve cancer vaccine responses as they target the suppressive tumor microenvironment, which limits CTL function [64,65]. Regulatory T cells can act negatively in the tumor, by blocking T helper cell and CTL functions through CTLA-4 [66,67]. ICI used against CTLA-4 can block these negative signals and enhance vaccine-induced tumor-specific CTL functions. PD-1 is an activation/inhibitory receptor that upregulates upon T cell activation but prevents T cell hyperactivation, regulating uncontrolled T cell function. However, in the tumor microenvironment it plays a critical inhibitory role in modulating the proliferation and cytolytic function of CTLs via its interaction with the ligand PD-L1, expressed on antigen-presenting and tumor cells. Antibodies blocking PD-1 prevent T cell exhaustion and assist in the proliferation of invigorated CD8 T cells from newly discovered TCF1 + CD8 T cells called stem-like cells [68]. Thus, PD1 blockade can not only invigorate but also protect vaccine-induced T cells from exhaustion in the tumor microenvironment, thereby prolonging antitumor activity of CTLs [69,70]. Thus, combining ICIs and therapeutic vaccines can be beneficial as they complement each other by favorably altering the immunosuppressive tumor microenvironment, increasing tumor immunogenicity and blocking negative regulations. However, for a positive clinical outcome, the type of vaccine and ICI to be used for each cancer type need to be carefully evaluated. Furthermore, the timing of vaccination in relation to ICI treatment and the sequence of both therapies needs to be standardized in preclinical models.

## 6. Safety and Efficacy of Cancer Vaccines in Combination with ICI

Immune-related adverse events (irAEs) are side effects of ICIs, and occur in 10–31% of treated patients with anti-PD-1 and anti-CTLA-4, respectively [71]. These irAE are mostly mediated by the activation of self-directed T cells or B cells, or the activation of macrophages and often resemble autoimmune diseases. They affect various tissues and organs, such as skin, gut, lungs, brain and endocrine organs. Anti-PD1 treatment seems to increase the risk for pneumonitis, hypothyroidism, myalgia and arthralgia, whereas for anti-CTLA-4 colitis and hypophysitis were more frequently described, and variations have been seen observed depending on the underlying tumor type [71,72]. Symptoms can be severe and are treated with immunosuppressants such as corticosteroids.

The combination of therapeutic cancer vaccines and ICIs have shown promising preclinical results and have not resulted in any concerns regarding toxicity. The administration of anti-PD-1 with GVAX vaccine increased survival and effective T cell response when compared to anti-PD-1 alone in a mouse model of pancreatic ductal adenocarcinoma [73]. Similarly, in a murine glioma model, the combination of a DC-based vaccine with anti-PD-1 prolonged survival compared to single treatments [74]. Similar responses were seen in a HPV-based viral vaccine and ICI [75]. Furthermore, the impact of a combination of anti-CTLA-4 antibodies and therapeutic cancer vaccines has been identified as a promising combination therapy, altering the balance of regulatory T cells by increasing effector T cells in tumors with enhanced anti-tumor function. [76,77,78]. The use of cancer vaccines and ICI in different clinical trials and their safety and efficacy has been well reviewed in [79]. Some of the completed clinical trials are summarized below in Table 2.

Therapeutic cancer vaccines as isolated therapy show modest clinical benefits, but if used in combination, they can generate tumor-specific immune responses associated with prolonged survival and improved clinical outcome. Responding patients show increased T cell infiltration in the tumor, and IFN-γ expression in T cells. These findings and many additional clinical trials support the idea that combining ICIs and cancer vaccines maximizes the potential of both treatments and that these combinations produce minimal additional irAEs compared to ICIs alone. Treatments can be further improved by associating ICIs with personalized cancer vaccines, due to their specificity and less off-target effects. A personalized neoantigen-based vaccine

NEO-PV-01, was used in combination with anti-PD-1 in a phase IIb clinical trial in patients with melanoma, non-small cell lung cancer (NSCLC), or bladder cancer. In a total of 82 patients, it was demonstrated that the regimen was safe, and no irAEs were reported. Antigen-specific CD4+ and CD8+ T cell with a cytotoxic phenotype were found in all patients after vaccination. These data support the safety and immunogenicity of combining neoantigens with ICIs, which is a promising strategy for the field of cancer vaccines [87]. Adequate controls in clinical trials and the selection of appropriate patient groups are important to allow maximal benefit from these combination therapies.

## 7. Vaccines against Infectious Agents in ICI-Treated Cancer Patients

Cancer patients are more susceptible to secondary infections compared to healthy individuals due to their immunosuppressive state, which is mostly caused by their treatment and oncological disease. Therefore, it is essential to protect these individuals from vaccine-preventable infections. For cancer patients receiving conventional chemotherapy, radiation therapy, or even autologous or allogenic stem cell transplantation, the use of non-live vaccines is widely recommended. Non-live vaccines such as the tetanus-diphtheria, influenza and the pneumococcal vaccine are safe for application in this population, but their efficacy is often reduced, necessitating serological follow-ups and additional vaccines doses. Live-attenuated vaccines, such as measles-mumps-rubella, chickenpox, yellow-fever or rotavirus vaccines are not used in cancer patients with active therapy given their impaired immunity, the risk of uncontrolled replication of attenuated vaccine strains and subsequent symptomatic vaccine-disease [88,89,90]. Interestingly, these vaccines are usually administered during childhood and rarely indicated in the adult population. The efficacy and safety of different vaccines in cancer patients is well reviewed elsewhere [91].

For patients receiving immunotherapy, however, there are gaps in knowledge regarding concomitant vaccine administration, especially because there are theoretical concerns that immune responses to vaccines could trigger irAEs. Only a few studies have assessed the safety and immunogenicity of vaccination during ICI treatment, and mainly influenza and SARS-CoV-2 vaccination have been investigated.

Influenza vaccines usually contain surface antigens without additional adjuvants, and are generally unlikely to be reactogenic and mostly elicit vaccine-specific humoral and CD4 T cell responses. Data on SARS-CoV-2 vaccines come mostly from considerably reactogenic mRNA vaccines that, in addition to humoral and CD4 T cell, induce CD8 T cell responses. Of note, there are some studies that use preventive to help cell-based immunotherapy. In a small study, patients received a tetanus booster to prime the immune system, enhancing the effects of a DC-based vaccine therapy against lethal brain tumors and dramatically improving patient survival and clinical outcome [92]. Similarly, in in vitro and animal studies rotavirus vaccine has been used to overcome ICI resistance [93].

### 7.1. Influenza Vaccines and ICIs

To date, most data on vaccination of ICI-treated patients that exist concern influenza vaccines, as cancer patients are at risk for severe influenza disease and are therefore usually vaccinated annually. After an initial small study (*n* = 23) with a retrospective unvaccinated control group (*n* = 40) suggested a potential increase in irAEs following vaccination [94], rising evidence that influenza vaccination in ICI-treated patients is safe and immunogenic has emerged [95]. This recent meta-analysis examined results from 10 studies, published between 2017 and 2020, and including a total of 1124 mostly anti-PD1-treated patients. A pooled total of 28.9% of vaccinated patients reported irAE, and the pooled incidence of high-grade toxicities (grade 3–4) in 697 patients was 7.5%. However, studies assessing the differences between vaccinated and unvaccinated patients were mostly retrospective [95]. Within this group, some studies showed comparable irAEs between vaccinated and nonvaccinated patients (37.4% vs. 42.6%, *p* = 0.06) [96], others found more irAEs in unvaccinated than vaccinated (36% vs. 55%; *p* = 0.1) [97], while others reported higher irAEs in vaccinated than in unvaccinated individuals [52% vs. 25.5% (Historical data)] [94]. Immunogenicity and the efficacy of influenza vaccination was reported in 986 patients, and although pooled analyses were impossible due to the heterogeneity of endpoints in the 8 studies, vaccination elicited antibody responses and had an overall effectiveness to prevent severe disease and hospitalization [95].

Interestingly, a recent multicenter Italian study from 2019/2020 compared the incidence and severity of influenza-like illness (ILI) in 581 vaccinated and 607 non-vaccinated individuals, however the vaccinated group was significantly older, had more comorbidities, especially cardiovascular and respiratory, and a poorer performance status. Nevertheless, while the incidence of ILI was similar in the vaccinated and unvaccinated group (*n*= 51 vs. *n*= 47), complications were significantly less frequent for vaccinated patients compared to unvaccinated patients (11.8% vs. 38.3% in unvaccinated, *p* = 0.002), and 2 unvaccinated patients died. In terms of safety, there were only few and quite mild vaccine-related irAEs. [98]. Taken together, these data suggest that influenza vaccination in patients under ICIs is safe and effective. However, further in-depth studies are required to analyze the vaccine-specific immune responses in patients that receive ICIs compared to those without ICI-treatment as to understand if ICI could potentially enhance vaccine immunogenicity and efficacy. The studies concerning influenza and COVID vaccines and ICIs are summarized in Table 3.

### 7.2. COVID-19 Vaccines and ICIs

The very recent development and widespread rollout of the COVID-19 vaccine has led to an exceptional interest in the understanding of vaccine safety, efficacy and immune responses in immunocompromised individuals, including patients under ICI treatment. Most COVID-19 vaccine studies in ICI populations used mRNA vaccines, and first results are now being published.

A study from Israel reported BNT162b2 (BioNTech/Pfizer) vaccination of 134 ICI-treated patients receiving 2 doses, who were age- and sex-matched with healthy controls. Vaccine-related side-effects were mostly comparable in both groups, and there was no exacerbation of pre-existing or new manifestation of immune-related side-effects, however, follow-up was limited to a median of 19 days after the second dose [99]. The safety profile in a Greek study in 59 anti-PD-1 or anti-PD-L1-treated patients found a single irAE during the median follow-up period of 44 days after a single vaccine dose of mostly mRNA (*n* = 44/59) and some viral vector vaccines (AZD1222, *n*= 15/59). Patients showed lower pseudo-neutralizing antibody titers 3 weeks after vaccination as compared to healthy controls (*n* = 283) [100]. However, a study from the US reported that anti-spike antibody titers were detectable in nearly all ICI-treated patients (97%, 30/31 patients) more than 7 days following mostly 2 doses of an mRNA vaccination [101].

Another study evaluated humoral and T cell responses in 88 patients, predominantly treated for lung cancer (76.1%) and under anti-PD-1 monotherapy (61.4%). A total of 78 patients received 2 doses of BNT162b2 (BioNTech/Pfizer) vaccine and 92% of patients had detectable IFN-γ T cell responses 3 weeks later. Almost all previously COVID-19-uninfected patients (95%) seroconverted for anti-spike antibodies and 77% had detectable neutralizing antibodies. In terms of safety, one patient reported two irAE, grade 3 hepatitis and colitis, in the 10 days following the first dose of vaccination [101]. Despite the limited sample sizes in studies evaluating safety and immunogenicity of COVID-19 vaccines in ICI-treated patients, these results are reassuring and encouraging, given the risk-benefit assessment of COIVD-19 vaccination in this vulnerable population. However, more studies and follow-up results are anticipated to further substantiate that vaccination in cancer patients under ICI is safe and effective.

## 8. Conclusions

Combining vaccination and ICIs can be beneficial for the efficacy of therapeutic cancer vaccines and appears to be safe, immunogenic end effective for conventional vaccines against infectious diseases. However, further in-depth studies are needed to analyze the immunological mechanisms of ICI treatments, with regard to the ways they can improve cancer vaccine efficacy, the timing of combining therapeutic vaccines and ICI and if this interaction can help expand the repertoire of cancer types that respond to ICI. In addition, long-term follow-ups to evaluate the longevity of immune responses and larger sample sizes from diverse populations are needed to account for individual variation in immune responses to vaccinations and ICIs.

## Figures and Tables

**Table 1:** Cancer vaccines and their clinical implementation. Summary of a selection of prophylactic and therapeutic vaccines in clinical trials or already in clinical use.

Cancer Type	Vaccine	Description	Type	Mechanism	Stage of Development	References
Bladder	BCG (Bacillus Calmette–Guérin)	Autologous (was used mainly against tuberculosis)	Therapeutic	Internalization of BCG antigen and activation of antigen-specific CD8 and CD4 T cells; direct cytotoxicity of the tumor cells	In clinical use	Reviewed in [21,22]
Brain	IDH1(R132H)-specific peptide vaccine	Allogenic (Isocitrate dehydrogenase1, gets mutated in gliomas)	Therapeutic	Specific immune response against the mutated protein	Phase 1	[23]
	DCVax^®^-L (Dendrtitic cell based personalized vaccine)	Autologous	Therapeutic	Patient-derived dendritic cells are labeled with patient’s tumor cells and injected intradermal to induce an immune response	Phase 3	[24]
Breast	Her 2 directed Cellular/DNA/viral	Autologous or allogenic	Therapeutic	Activation of immune response	Phase 1/2	Many Phase1/phase 2 trials reviewed in [25]
	h tert (telomerase reverse transcriptase)	Autologous or allogenic	Therapeutic	Activation of CTLs against mutations in overexpressing breast cancer cells		Reviewed in [26].
Prostate	Sipuleucel-T (prostate acid phosphatase antigen (PAP))	Autologous (patients APCs incubated with PAP and GM-CSF)	Therapeutic	T cell	In clinical use	[27,28]
Colorectal	CEA (carcinoembryonic antigen) Muc1 Peptide/DNA	Autologous Allogenic Allogenic	Therapeutic	CTL response	Preclinical, In clinical trial	[29,30,31]
Kidney	carbonic-anhydrase IX HLA-A 0201/0206-restricted epitope peptide (HIG2-9-4) vaccine	Allogenic/ Autologous Autologous	Therapeutic Therapeutic	Increase in IFN responses CTL CTL	In clinical trials	[32,33,34]
Liver	HEPLISAV-B Heptatis B surface antigen	Allogenic	Preventive	antibody response CTL	In clinical use	[35]
Lung	CIMAvax-EGF	Allogenic	Preventive	antibody	Clinical trial	[36]
Melanoma	Neovax (personalized neoantigens)	Autologous	Preventive (after surgery)	CD4 and CD8	Clinical trial	[37]
Cervical	Gardasil4/9 Cervarix–contain L1 proteins from different strains	Allogenic	Preventive	Mainly induces neutralizing antibodies against various strains of HPV	In clinical use	[38,39]

**Table 2:** Combination therapies of cancer vaccine and ICIs in clinical trials. irAE: immune-related adverse events.

Cancer Type	Vaccine	ICI Agent	Type	Summary	Reference
**Melanoma**	Talimogene Laherparepvec (GM-CSF)	Pembrolizumab	Phase III	Well tolerated (only grade 1 and 2 toxicities) and showed OR = 62%	[80]
**Head and Neck**	Talimogene Laherparepvec (GM-CSF)	Pembrolizumab	Phase 1	irAEs > 50%, related to either GM-CSF or Pembrolizumab Only 13% partial response	[81]
**Melanoma**	gp100280-288 (288 V), and NY-ESO-1157-165 (165 V). peptide vaccine	Nivolumab	Phase 1	Well tolerated: 53% had disease progression at 2 years. Progression was associated with increased regulatory T cells and a decrease in antigen-specific CD8 T cells	[82]
**Melanoma**	Talimogene Laherparepvec (GM-CSF)	Ipilimumab	Phase 1	Well tolerated, Grade 3 and 4 irAEs: 26%. Objective response: 50%	[83]
**Prostate cancer**	Sipuleucel-T (SIP-T)	Ipilimumab	Phase III	Adverse effects negligible. Median survival < 4 years	[84]
**Prostate cancer**	GVAX	ipilimumab	Phase I	Well tolerated. 50% reduction in prostate-specific antigen in the combination group	[85]
**Metastatic melanoma**	Gp100 peptide vaccine	Ipilimumab	Phase III	Well tolerated. Grade 3 and 4 irAEs: 10–15%. Overall survival: 10 months for combination vs. 6 months for GP100 alone. No difference between ipi and combination	[86]
**Melanoma/bladder and lung cancer**	Neo-PV-01 (personalized neoantigens)	Nivolumab	Phase IIb	Safe, efficacious and activation of CD4 and CD8 T Cells	[87]

**Table 3:** Summary of irAEs and immune responses after vaccinations in cancer patients undergoing ICI.

Cancer Type	Vaccine	Patient Number	Safety	Efficacy	Reference
Lung	Influenza	Vaccinated; *n* = 23 Healthy: *n* = 11 control cancer: *n* = 40	irAEs 52.2% vs. 25.5%	Similar humoral response in healthy or cancer patients	[94]
Different cancer types	Influenza	*n* = 1124	Any grade irAEs 28.9%, grade 3–4: 7.5%	High antibody titers, CTLs, very few patients experienced Influenza infection	[95]
Different cancer types	Influenza	*n* = 1188 vaccinated *n* = 581 unvaccinated *n* = 607	Vaccine related adverse events: 1.5% grades 1–2	Similar incidence of influenza-like illness, Fatality 4.3% (unvaccinated) vs. 0% (vaccinated)	[98]
Many types, 50% lung cancer	COVID-19	*n* = 134	No Vaccine related toxicities	NA	[99]
Different cancer types; Lung and bladder 25% each	COVID-19	*n* = 59 (cancer patients) *n* = 283 (controls)	Only one patient had irAEs	Neutralizing antibody titers 22% vs. 38% in controls	[100]
Different types; Lung cancer 76%	COVID-19	*n* = 88	Fever and pain at injection site. 1 patient with grade 3 irAEs.	High seropositivity, and CD8 and CD4 responses	[101]

## Data Availability

No data was analyzed or generated in this study.
